# An Improved Validated Method for the Determination of Short-Chain Fatty Acids in Human Fecal Samples by Gas Chromatography with Flame Ionization Detection (GC-FID)

**DOI:** 10.3390/metabo13111106

**Published:** 2023-10-24

**Authors:** Morganne Smith, Lee Polite, Andreas Christy, Indika Edirisinghe, Britt Burton-Freeman, Amandeep Sandhu

**Affiliations:** 1 Department of Food Science and Nutrition and Center for Nutrition Research, Illinois Institute of Technology, Chicago, IL 60616, USA; mfreema8@iit.edu (M.S.); iedirisi@iit.edu (I.E.); bburton@iit.edu (B.B.-F.); 2 Axion Analytical Labs Inc., Chicago, IL 60607, USA; lee@axionlabs.com (L.P.); ac2671@pcom.edu (A.C.)

**Keywords:** short-chain fatty acids (SCFAs), gas chromatography, flame ionization detection, GC-FID

## Abstract

Short-chain fatty acids (SCFAs) are metabolites produced by the gut microbiota through the fermentation of non-digestible carbohydrates. Recent studies suggest that the gut microbiota composition, diet and metabolic status play an important role in the production of SCFAs. The primary objective of this study was to develop a simplified method for SCFA analysis in human fecal samples by gas chromatography with flame ionization detection (GC-FID). The secondary objective was to apply the method to fecal samples collected from a clinical trial. The developed GC-FID method showed excellent linearity (R^2^ > 0.99994), with a limit of detection (LOD) ranging from 0.02 to 0.23 µg/mL and a limit of quantification (LOQ) ranging from 0.08 to 0.78 µg/mL. Recovery for the method ranged between 54.24 ± 1.17% and 140.94 ± 2.10%. Intra- and inter-day repeatability ranged from 0.56 to 1.03 and from 0.10 to 4.76% RSD, respectively. Nine SCFAs were identified and quantified (acetic, propionic, iso-butyric, butyric, iso-valeric, valeric, 4-methyl valeric, hexanoic and heptanoic acids) in freeze-dried fecal samples. The clinical trial compared participants with prediabetes mellitus and insulin resistance (IR-group, *n* = 20) to metabolically healthy participants (reference group, R-group, *n* = 9) following a 4-week intervention of a daily red raspberry smoothie (RRB, 1 cup fresh-weight equivalent) with or without fructo-oligosaccharide (RRB + FOS, 1 cup RRB + 8 g FOS). The statistical analysis (Student’s *t*-test, ANCOVA) was performed on PC-SAS 9.4 (SAS Institute). Acetic acid was higher in the R-group compared to the IR-group at baseline/week 0 (*p* = 0.14). No significant changes in fecal SCFA content were observed after 4 weeks of either RRB or RRB + FOS.

## 1. Introduction

Short-chain fatty acids (SCFAs) are metabolites produced by the gut microbiota through the fermentation of non-digestible carbohydrates. The gut microbiome is comprised of several trillion bacteria and other microorganisms with distinctive capabilities. These microbes play a variety of important roles, such as maintaining the integrity of colon epithelial cells, protecting from pathogens, providing vitamins and synthesizing metabolic compounds that benefit humans. When available, SCFAs are estimated to provide up to 10% of the caloric intake of humans [1]. In addition, SCFAs are present in different amounts depending on an individual’s health status. Lower quantities of SCFAs have been found in patients with intestinal diseases (i.e., irritable bowel disorder and colorectal cancer), exemplifying their effectiveness as markers of health [2].

The “short-chain” classification is derived from the number of carbons, which typically range from one to six. The most abundant SCFAs in human feces are acetic (C2), propionic (C3) and butyric (C4), consisting of about 95% of SCFA content in a ratio of 60:20:20, respectively [3]. Other SCFAs found in lower quantities, specifically those with branched chains, are produced through protein metabolism [4]. These include iso-butyric (C4), valeric (C5), iso-valeric (C5) and 4-methyl valeric (C6). Hexanoic (C6) and heptanoic (C7) acids have previously been reported in clinical trial studies; however, their production and utility in the human body are less certain [5].

In general, research on the production and absorption of SCFAs has focused on the three most abundant acids: acetic, propionic and butyric. All three acids are produced through the glycolysis pathway of carbohydrate fermentation in bacteria, although it has been reported they can also be produced as by-products of protein fermentation [4]. Pyruvate is the main product of glycolysis, which is converted into acetic, propionic and butyric acids through various pathways. In the production of acetic acid, pyruvate is converted to acetyl-CoA through the pyruvate dehydrogenase complex. Acetyl-CoA is then converted to acetate by microbial-specific enzymes: phosphotransacetylase and acetate kinase [6]. Propionic acid is formed through the Wood–Werkman Cycle, which incorporates the conversion of oxaloacetate to succinate in the Krebs cycle. Once succinate is formed, it is then converted into propionic acid via decarboxylation. Propionic acid may also be produced through the acrylate pathway, where acryloyl-CoA mediates the reduction of lactate to propionic acid [7]. Similar to acetic acid, butyric acid is produced by the conversion of acetyl-CoA to butyryl-CoA via microbial butyrate kinase and butyryl-CoA: acetyl-CoA transferase [3,8]. The production of SCFAs with C5 to C7 compositions is less understood.

There are only a few validated methods for SCFA analysis, and their application to human biological samples is limited. Fecal samples are considered a complex sample matrix. Traditional methods of SCFA analysis emphasize sample preparation techniques to achieve a “cleaner” sample before analysis by analytical instrumentation [9]. These methods range in complexity from vacuum or steam distillation to extraction with various solvents or simple filtration methods, each providing its own challenges and benefits. Distillation techniques are time-consuming compared to simpler methods for clean-up. Yet, filtration and centrifugation have the potential to contain high numbers of impurities in samples prepared for analysis. Tert-butylmethyl ether (TBME), diethyl ether and pentafluorobenzyl bromide (PFBBr) are solvents that have previously been used for sample preparation, though they impose safety hazards and increased costs to the method [2,10,11,12]. Additional techniques such as derivatization by 3-nitrophenylhydrazine, N,O-Bis(trimethylsilyl)trifluoroacetamide (BSTFA) or chloroformate with isobutanol have been used but impose similar challenges, as previously described [13,14].

The current literature lacks quick and simple standardized methods for SCFA analysis in human fecal samples. Therefore, the primary objective of this study was to develop and validate a simplified method for SCFA extraction and analysis using GC-FID. This method focused on eliminating the use of derivatization and distillation techniques and mitigating the use of harsh chemicals. A secondary objective was to apply the validated method to fecal samples from a previously conducted clinical trial (Clinicaltrials.gov NCT03049631, IRB#2016-136) to verify its utility in human research. The clinical protocol was a 4-week dietary intervention aimed at influencing the gut microbiota composition in adults with and without prediabetes and insulin resistance [15,16]. The clinical trial fecal samples were used: (1) to characterize the SCFA content in metabolically discrete groups, (2) to assess the effects of the dietary intervention on the SCFA content in fecal samples and (3) to discuss fecal SCFA content as a useful biomarker for gut function and composition.

## 2. Materials and Methods

### 2.1. Research Design

For objective one, the initial method development procedures compared two sample extraction solvents: TBME with a liquid–liquid extraction method and a simple water-based aqueous extraction (AE) method. The TBME method served as the comparator for the developed AE method. The goal was to simplify sample preparation, aiming for a “dilute-and-shoot” type method that would maintain sample integrity while reducing the use of organic solvents. 

For objective two, biological samples from a previous clinical trial (Clinicaltrials.gov NCT03049631, IRB#2016-136) were used. The study design and results have been published elsewhere [15,16]. Briefly, thirty-six adult men and women participated in a randomized dietary intervention study. Twenty-six individuals met the criteria for prediabetes with insulin resistance (IR-group). Ten were metabolically healthy individuals (R-group). The participants consumed red raspberry (RRB, 1 cup fresh-weight equivalent) or red raspberry with fructo-oligosaccharide (RBB + FOS, 1 cup RRB + 8 g FOS) for 4 weeks each, with a 4-week washout between intervention periods. Fecal samples were collected at baseline (week 0) and 4 weeks. The participants’ fecal samples used in the current study were from those people who provided written informed consent to use their collected samples for future research (*n* = 20 for the IR-group and *n* = 9 for the R-group). The samples were stored at −80 °C until they were processed for analysis. 

### 2.2. Chemicals and Reagents

Analytical grade standards (acetic, propionic, iso-butyric, butyric, iso-valeric, valeric and heptanoic acids) were purchased from Millipore Sigma (St. Louis, MO, USA), except for 4-methyl valeric and hexanoic acid, which were purchased from Fisher Scientific (Hampton, NH, USA). Internal standard (IS) 2-ethylbutyric acid was purchased from TCI America (Portland, OR). Tert-butyl methyl ether and sodium bicarbonate were purchased from Millipore Sigma (St. Louis, MO, USA). MilliQ water was obtained from a Direct-Q Water Purification System (18.2 MΩ-cm at 25 °C, Millipore Sigma, St. Louis, MO, USA).

### 2.3. GC-FID Method

The analysis of the SCFAs was performed on an Agilent 7890A GC-FID equipped with an Agilent J&W GC Column DB-FFAP (30 m × 0.25 mm × 0.5 µm). The Agilent OpenLab ChemStation version B.04.03 was used for data collection. The injection temperature was 300 °C. Samples were injected in pulsed split injection mode in 1 µL aliquots. The initial oven temperature was 100 °C, with a hold time of 0 min. The oven temperature increased from 100 °C to 250 °C at 20 °C/min, with a final hold time of 0 min. Helium was the carrier gas, with a flow rate of 1.7682 mL/min. The total run time of the method was 10 min. 

### 2.4. Standard Preparation and Method Validation

A stock standard solution containing all nine target SCFAs was prepared in 50/50 acetonitrile: water (ACN:H_2_O). The stock solution was diluted in ACN:H_2_O to concentrations ranging from 5 to 2000 ppm, acidified with 5.0 M HCl and spiked with an internal standard (2-ethyl butyric acid) to a final concentration of 1000 ppm. A six-point calibration curve was created for standards at the following concentrations: 5, 50, 500, 750, 1000 and 2000 ppm (Table 1). Each standard was injected in triplicate. The limit of detection (LOD) and limit of quantification (LOQ) were calculated for each SCFA using signal-to-noise ratios of 3 for LOD and 10 for LOQ, respectively. Within a day (intra-day) and between days (inter-day) variability was determined by six standards, excluding iso-butyric acid due to the limited resource availability. Intra-day precision was determined by an analysis of the 2000 ppm standard repeated three times on a single day, each time in triplicate. Inter-day precision was determined by an analysis of standards at 50, 750 and 2000 ppm over three consecutive days. Due to the complex matrix of the fecal samples, recovery was performed using solvent method blanks. Method blanks were spiked with 100 or 1000 ppm of a standard mix containing all SCFAs (except iso-butyric acid) and prepared by the AE method.

### 2.5. Sample Preparation for Extraction

Methods were developed for the extraction of SCFAs using a homogenized fecal sample from a single participant (IRB#2016-136). Before extraction, the sample was thawed and weighed to about 1.0 g of fresh weight in triplicate and spiked with an internal standard to achieve a final concentration of 1000 ppm. 

#### 2.5.1. Tert-Butyl Methyl Ether Extraction (TBME) Method

The traditional ether extraction method for comparisons was derived from Niccolai et al. with minor modifications [2]. A 1:1 *w*/*v* equivalent of 10 mM sodium bicarbonate buffer was added to the weighed fresh sample (FR) and vortexed for 30 s. The sample was then placed in an ultrasonic bath for 5 min and subsequently centrifuged at 3214× *g* for 10 min. The supernatant was collected and transferred to a 1.5 mL plastic microcentrifuge tube. Then, a 100 µL aliquot of the collected supernatant was added to a separate 1.5 mL plastic microcentrifuge tube containing 1 mL of TBME and 50 µL of 1.0 M HCl acid. The mixture was vortexed for 2 min and then centrifuged at 12,857× *g* for 5 min. Liquid–liquid extraction was performed, and the solvent layer was transferred to an autosampler vial for GC-FID analysis. 

#### 2.5.2. Aqueous Extraction (AE) Method

MilliQ water (5 mL) was added to the weighed sample (1.0 g, FR), and the sample was vortexed for 3 min. The homogenized mixture was placed in an ultrasonic bath for 5 min and then centrifuged at 3214× *g* for 10 min. The supernatant (~4 mL) was collected and transferred into a clean 15 mL falcon tube, where 200 µL of 5.0 M HCl acid was added to the sample. The sample was left to rest for 10 min with occasional shaking and then centrifuged at 12,857× *g* for 5 min. The acidified, aqueous supernatant was transferred to an auto sampler vial for GC-FID analysis.

#### 2.5.3. Lyophilization Pre-Treatment

To gain an understanding of the influence of the moisture content on the fecal SCFA analysis, lyophilized/freeze-dried samples (FD) were compared to fresh samples (FR). The fecal samples were obtained from a single participant. The frozen sample was thawed and weighed to about 1.0 g for FR sample preparation. The remaining sample was lyophilized for 24 h at −87 °C and 32 mT (Millrock BenchTop Manifold Freeze-Drier BT85, Millrock Technology, Inc., Kingston, NY, USA). The samples were processed immediately after 24 h of lyophilization. The FD sample was weighed to about 0.5 g for sample preparation. Both FR and FD samples were extracted for SCFAs by the AE method in triplicate. 

### 2.6. Statistical Analysis

Statistical analysis was performed on PC-SAS 9.4 (SAS Institute). The normality distributions were analyzed by the Shapiro–Wilk test (*p* > 0.05 was considered a normal distribution). All data had acceptable distributions. Student’s *t*-test was used to test the differences between the AE and TBME extraction methods and FD and FR samples. Statistical significance was determined based on a two-sided comparison at the 5% significance level (*p* < 0.05). Student’s *t*-test analysis was also used to determine differences between week 0 (WK0)- and week 8 (WK8) samples to assess the effect of washout. No statistical difference was found between WK0 and WK8 for any SCFAs; therefore, WK0 was used as the baseline. Analytes that did not conform to normal distributions were log_10_-transformed prior to the statistical analysis. Student’s *t*-test on log_10_-transformed data was used to test the metabolic status of SCFA outcomes. Mixed model analysis of covariance (ANCOVA) was performed on each dependent variable (individual SCFAs), including total SCFA content, to test the main effect of the intervention (RRB vs. RRB + FOS) after 4 weeks, with the baseline as the covariate and the participant as the random variable. Age, gender and ethnicity were tested for significance in each model and included when significant. Statistical significance was determined based on a two-sided comparison at the 5% significance level (*p* < 0.05) under a null hypothesis of no difference in metabolic status and no difference between interventions.

## 3. Results

### 3.1. GC-FID Method Validation

Nine SCFAs were characterized in the development of this method. The relevant SCFAs are listed in order of their retention times in Table 1. The linearity of the calibration data is represented by regression coefficients (R^2^) for each SCFA. The regression coefficients ranged from 0.99994 to 0.99998. The LODs ranged from 0.02 to 0.23 µg/mL, and the LOQs ranged from 0.08 to 0.78 µg/mL. The precision data ranges for each SCFA are represented as percent relative standard deviations (% RSD) in Table 1. The intra-day precision at the 2000 ppm standard level ranged from 0.56 to 1.03% RSD among the nine SCFAs. The inter-day % RSD data ranged from 0.45 to 4.76% at the 50 ppm level, from 0.10 to 1.52% at the 750 ppm level and from 0.18 to 0.93% at the 2000 ppm level. Recovery was conducted by spiking solvent method blanks with 100 or 1000 ppm of a standard containing all SCFAs except iso-butyric. Table 2 shows recovery percentages ranging from 57.64 ± 2.89% to 128.97 ± 5.43% at the 100 ppm level and from 54.24 ± 1.17% to 140.94 ± 2.10% at the 1000 ppm level.

### 3.2. Sample Preparation

The AE and TBME methods were compared based on concentrations of SCFAs, including the calculated % RSD of the two methods. The data analysis accounted for corrections for the sample weight and total volume. The concentrations of each SCFA are significantly higher (*p* < 0.0001) in the AE method as compared to those in the organic solvent extraction method (TBME), indicating improved sensitivity with AE extraction (Table 3, Figure S1). The % RSDs also showed an improved reproducibility of the extraction of SCFAs using the AE method compared to TBME, as indicated by less variation with AE vs. TBME (Table 3). For these reasons, the AE method was selected as the extraction method for fecal sample preparation from the clinical trial. Though the AE method improved sensitivity and reproducibility compared to the TBME, the water content of the fecal samples may contribute to variance. Water content of fecal samples vary within and between individuals. Lyophilization is a common technique used to remove water content while maintaining the integrity of the sample matrix. Hsu et al. observed decreased variance in SCFAs in lyophilized fecal samples [17]. The lyophilization technique was explored for the current method and compared against FR samples to determine the effects on variance. 

FR samples were converted to dry-weight equivalents for comparison purposes. No significant difference was found between the methods (FR vs. FD, *p* > 0.05) in all SCFAs except for 4-methyl valeric acid (*p* = 1.62 × 10^−5^) and heptanoic acid (*p* = 0.011, Table 4, Figure S2). The significance for 4-methyl valeric and heptanoic acids was driven by the ability to detect these SCFAs in FD samples compared to FR samples. Lyophilization improved sensitivity by concentrating the sample. It is important to note that this was conducted as a within-participant experiment on a single collection day. For a larger-scale study, the water content of stool samples will vary between participants as well as within individual participants at each collection time point. Therefore, lyophilzation is recommended to reduce this variance between and among participants. Overall, the developed method provides a shorter run time, improved linear regression, less variance, improved sensitivity and lower LOD and LOQ values compared to previous methods [18,19].

### 3.3. Application of the Validated Method to Fecal Samples Collected from a Previous Clinical Trial

#### 3.3.1. Participant Demographics

The selected population for the study included a total of 29 male and female adults (IR-group *n* = 20; R-group *n* = 9). The baseline demographics are shown in Table 5.

#### 3.3.2. Assessment of Metabolic Status on SCFAs

**Figure 1:**
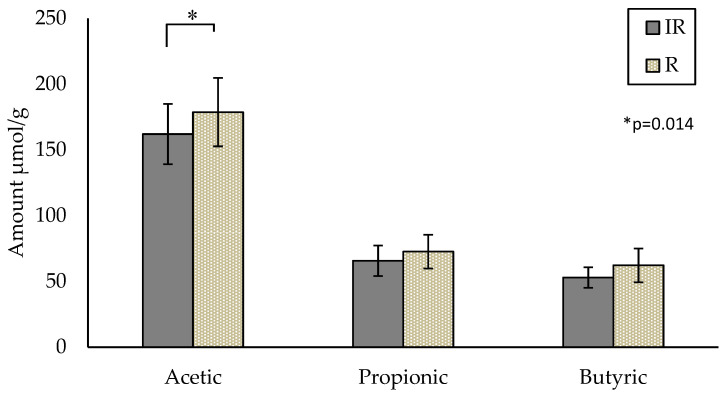
Comparison of three major SCFAs at baseline (Week 0) between the IR-group (*n* = 20) and R-group (*n* = 7) (*p* = 0.014). Values are unadjusted means ± SEM; IR—insulin-resistant; R—reference.

The SCFA content analyzed by the AE method provided results consistent with the available literature [20]. Acetic acid is the predominant SCFA, followed by propionic and butyric acids. Verbeke et al. reported concentrations ranging from 35.8 ± 2.4 to 320.3 ± 24.9 µmol/g for acetic acid and from 11.4 ± 1.2 to 97.3 ± 10.5 µmol/g and from 8.8 ± 5.2 to 93.8 ± 9.13 µmol/g for propionic and butyric acid, respectively [20]. The mean values from both groups in the current study are within the range of the relevant literature. The participant mean values at baseline (WK0) for acetic, propionic and butyric acids were 162.0 ± 22.9, 65.7 ± 11.7 and 52.9 ± 7.8 µmol/g for the IR-group and 178.7 ± 26.0, 72.6 ± 12.9 and 62.2 ± 12.9 µmol/g for the R-group, respectively (Figure 1). A significant difference in acetic acid (*p* = 0.014) was observed between the IR-group compared to the R-group at baseline (Week 0) only (Figure 1). Two outliers were identified by box-plot outlier analysis in the R-group and were removed for the analysis. Propionic and butyric acids show similar trends (*p* = 0.087, *p* = 0.078, respectively), with the R-group containing higher amounts of each. 

#### 3.3.3. Assessment of Intervention on SCFAs 

Metabolic groups were analyzed separately due to differences at baseline. Moderate downward shifts in the SCFA content after 4 weeks of RRB and RRB + FOS intervention were observed in both groups (Table 6). A significant difference was observed in 4-methyl valeric acid (*p* = 0.040) in the R-group after the intake of study interventions (Table 6). No other statistically significant differences (*p* > 0.05) were observed in SCFAs between interventions in either group (Table 6). 

The current study developed and applied a simplified method to measure SCFAs in human fecal samples using GC-FID. We observed improved sensitivity and reproducibility in an aqueous extraction method (AE) compared to a typical SCFA extraction method using organic solvents (TBME) (Table 3). The addition of lyophilization for sample preparation further improved the sensitivity of SCFA analysis by GC-FID. The new simplified method was applied to human fecal samples collected from individuals with and without prediabetes and insulin resistance who participated in a clinical trial. The results indicated significantly lower acetic acid in the IR-group compared to the R-group. A modest 4-week dietary intervention with red raspberry and red raspberry plus FOS supplementation had a limited influence on the SCFA content. 4-methyl valeric acid in the R-group was significantly lower after the intervention, while all other changes in SCFAs were non-significant. 

## 4. Discussion

### 4.1. Method Development and Validation

Linear regression data as well as LOD, LOQ and precision metrics are reported in Table 1. The method showed excellent linearity parameters, with R^2^ values greater than 0.99994. The LOD and LOQ ranged from 0.02 to 0.23 µg/mL and from 0.08 to 0.78 µg/mL, respectively. Comparatively, Scortichini et al. reported R^2^, LOD and LOQ ranges from 0.9989 to 0.9998, from 0.04 to 0.64 µM (corresponding to 0.004 to 0.038 µg/mL) and from 0.14 to 2.12 µM (corresponding to 0.012 to 0.127 µg/mL), respectively [11]. Likewise, Zhao et al. reported LOD and LOQ ranges from 0.72 to 9.04 µM (corresponding to 0.083 to 0.543 µg/mL) and from 2.38 to 30.14 µM (corresponding to 0.276 to 1.081 µg/mL), respectively [19]. In the current method, the intra- and inter-day precision ranged from 0.56 to 1.03 (%RSD) and from 0.18 to 0.93% in the 2000 ppm standard. The inter- and intra-day precision reported by Scortichini et al. was assessed using 20 mg of fecal sample and ranged from 1.2 to 4.6% and from 0.6 to 4.2% [11]. Zhao et al. assessed inter- and intra-day precision in a standard solution containing SCFAs at varied concentrations. They reported inter- and intra-day ranges between 1.51 and 10.32% and between 1.41 and 5.98%, respectively [19]. The overall recovery percentages for the current method ranged from 54.24 to 140.94%. Zhao et al. reported recovery values from 87.7 to 114.5% for acetic, propionic, iso-butyric, butyric, iso-valeric and valeric acids, citing caproic (hexanoic) and heptanoic as acids of concern, with a low recovery between 61.1 and 85.9% and between 45.5 and 51.3%, respectively [19]. Acetic and heptanoic acids were the acids of concern for the current method, with the lowest and highest recovery percentages of all the SCFAs (Table 2). Excluding these SCFAs, the range of recovery for the current method would be 87.60 to 129.74%, which is congruent with the relevant literature.

### 4.2. Understanding Metabolic Status and SCFAs

The gut microbiome is central to various components of human health and the maintenance of homeostasis. However, dysbiosis of the gut microbiome has been shown to influence immune system function, the central nervous system and have a role in metabolic disorders as well as gastrointestinal diseases and conditions [21,22]. These disease states and conditions have also been shown to impact the SCFA content. Niccolai et al. showed a significantly lower total SCFA content in participants with colorectal cancer compared to that in a healthy population, while a group of people with celiac disease showed a greater SCFA content compared to the healthy population, though not statistically significantly [2]. De la Cuesta-Zuluaga et al. also reported positive associations between obesity, central obesity and hypertension and SCFA content [23]. In the present study, variations in SCFAs were noted between metabolically distinct groups (IR vs. R). Associations between SCFAs and insulin resistance are limited, though Weitkunat et al. demonstrated significantly improved insulin sensitivity in diet-induced obese rats after a 6-week high-fat diet intervention supplemented with propionate compared to a high-fat control diet [24]. Similar trends were observed with a high-fat diet supplemented with acetate, though they were not significant [24]. Gao et al. showed similar effects on insulin sensitivity in mice with butyrate supplementation in a high-fat diet [25].

The current study aimed to understand volatile SCFAs as a by-product of microbial fermentation in different metabolic states reflecting microbiota composition and function. Among the three major SCFAs, the IR-group had lower acetic, propionic and butyric acid contents compared to the R-group at baseline (*p* = 0.014, 0.087 and 0.078, respectively). A significant shift in 14 species of gut bacteria between the IR-group and R-group was observed in our previous report on gut microbiota composition [15]. A greater abundance of *Blautia obeum*, *Blautia wexlerae*, *Clostridium clostridioforme* and *Ruminococcus gnavus* and fewer *Bacteroides dorei*, *Coprococcus eutactus*, *Eubacterium eligens* and *Bacteroides eggerthii* were identified in the IR-group compared to the R-group [15]. An in vitro study reported that acetate production by *E. eligens* was associated with the metabolism of pectin [26]. Our previous gut microbiota work showed the R-group to have a greater abundance of *E. eligens* species at baseline compared to the IR-group [15]. Likewise, the current report showed the R-group had more acetic acid content at baseline. The greater abundance of *E. eligens* within the R-group may be a contributing factor to the higher acetic acid levels that were observed in this group. These initial findings suggest a relationship between metabolic status and gut microbiota composition, with a potential role of SCFAs.

### 4.3. Response to Study Intervention

The diet acts as a modulator of gut microbiota composition and function. Dietary components that feed and promote the growth of beneficial gut bacteria are called prebiotics. Dietary fibers and certain (poly)phenols promote the growth of beneficial bacteria. Likewise, the metabolic capabilities of the gut microbiota enable the bioavailability of nutrients and other bioactive compounds to the human host that might not otherwise be accessible. Zheng et al. demonstrated that FOS and (poly)phenols modulate the gut microbiome of rats by altering the microbiota composition and associated SCFAs [27]. They compared a high-FOS diet to a high-FOS diet supplemented with either quercetin (FOS-Q) or catechin (FOS-C) in rats after a 4-week period. Their findings suggested that FOS-Q promoted the growth of *Firmicutes*, typically associated with butyrate production [27]. Correspondingly, FOS-Q increased total SCFA production by elevating levels of propionic and butyric acid compared to a high-FOS diet alone. FOS-C, on the other hand, significantly increased propionic acid production; however, total SCFA production was lower than that of both the high-FOS diet alone and FOS-Q due to reduced levels of acetic and butyric acids. The results observed by Zheng et al. highlight the effects of pairing different (poly)phenols with FOS on the gut microbiome and related metabolites [27]. The current study tested the effects of a chronic intake of a (poly)phenol-rich smoothie (RRB) compared to one supplemented with fiber (RRB + FOS). Red raspberries are high in anthocyanins and ellagitannins [15]. The results of the current study and those observed by Zheng et al. indicate a possible interaction between FOS and (poly)phenols and their activity towards the gut microbiome and resulting fecal SCFA content. It is noteworthy that studies in humans with FOS interventions at dosages from 5 to 30 g have reported no change in total fecal SCFAs [28,29]. The FOS dose in the present study was 8 g, on the lower side of the previous literature, which would further support our findings of no change in the SCFA content.

A significant difference in 4-methyl valeric acid (*p* = 0.040) was observed in the metabolically healthy reference group (R-group, *n* = 9) when comparing RRB to RRB + FOS at Week 4 (Table 6). Limited information is available for the production of 4-methly valeric acid in humans, though as a branched-chain SCFA, it could be produced by protein fermentation [4]. In this case, the background diet should be considered as a contributing factor to the SCFA content. Similarly, the sample size for the R-group was relatively small and should be considered when interpreting the results.

### 4.4. Metabolic Fate of SCFAs

Gut bacteria range in their ability to produce and consume SCFAs. The ability of *Roseburia* spp. to produce butyrate while consuming acetate exemplifies this trait [30]. SCFAs are involved in several other mechanisms. Colonocytes utilize butyrate as the main source of fuel, in which their consumption may be rapid depending on the energy needs of epithelial cells [31]. Other SCFAs enter portal circulation, where they travel to the liver and are involved in inflammatory response mechanisms and/or metabolic regulation. In addition, SCFAs may enter systemic circulation and are detected in the blood, albeit at much lower concentrations than detected in fecal samples [32]. The current study showed limited changes in the SCFA content after the dietary interventions. There are multiple pathways and uses of SCFAs by individuals based on their metabolic and physiological status. Likewise, the balance of the production, absorption and excretion of SCFA should be considered. The current study does not provide data on these parameters; however, the microbiome and metabolic results of the parent study showed changes in select gut microbiota, improved hepatic insulin sensitivity and reduced lipids with RRB and RRB + FOS supplementation [16]. The clinical effects are consistent with actions of SCFAs, suggesting possible increased production and absorption resulting in relatively stable luminal concentrations. Ideally, gut bacteria work symbiotically with their human hosts to provide the necessary SCFAs to maintain homeostasis in the human body while retaining SCFAs required by the microbes to flourish. In a state of dysbiosis in the gut microbiome, resource delegation may change and, likewise, the fate of SCFAs may change too. The current study utilized only human fecal samples to analyze SCFAs and did not observe any differences in the total SCFA content with dietary intervention. Multiple biological samples (blood, urine and fecal) may be required to understand the allocation of SCFAs in both healthy and diseased states.

## 5. Conclusions

The proposed sample extraction method and analysis by GC-FID were validated for the quantification of SCFAs in human fecal samples. The simplified method improves the sensitivity and reproducibility of SCFA analysis without using organic solvents. Additional advantages of this method compared to previously published work include eliminating complex and time-consuming techniques such as derivatization and distillation. Some of the drawbacks of the proposed method included the intricacies of the sample matrix and the low concentrations of certain SCFAs; however, these are common issues faced with SCFA analysis methods. Lyophilization was included in this method to help with water variability and matrix challenges.

The current study showed that the baseline concentration of acetic acid was significantly different between metabolically distinct individuals (IR-group vs. R-group). In addition, a significant difference (*p* = 0.040) was observed in the 4-methyl valeric acid content in R-group after a 4-week intervention of RRB compared to RRB + FOS, but no other significant differences in SCFAs were observed. The interplay of the SCFAs, gut microbiome, diet and metabolic status is complex. The gut microbiome serves as the central organ system connecting dietary influences, metabolic health and local and systemic SCFA content. In the present study, adding (poly)phenols (RRB) and two sources of fiber (RRB + FOS) to the diet daily did not alter the concentrations of SCFAs in fecal matter. However, the original study did reveal changes in the composition of gut bacteria and clinical end points [16]. The shifts in gut microbiota, specifically in SCFA producers and consumers, suggest that there may be additional mechanisms at work in the metabolism of SCFAs. Future research in SCFA analysis should be directed toward understanding these mechanisms of action.Multiple biological samples, such as the combination of blood, urine and fecal samples, may be necessary to understand SCFA production, absorption and utilization by humans.

## Figures and Tables

**Table 1:** Linear regression data from a six-point calibration curve and parameters of method validation.

SCFA	Retention Time (min)	Slope (m)	Intercept (*b*)	R^2^	LOD (µg/mL)	LOQ (µg/mL)	Intra-Day at 2000 ppm (% RSD)	Inter-Day at 50 ppm (% RSD)	Inter-Day at 750 ppm (% RSD)	Inter-Day at 2000 ppm (% RSD)
Acetic	3.32	0.04	0.002	0.99994	0.15	0.50	0.66	4.76	0.92	0.35
Propionic	3.74	0.61	0.001	0.99996	0.17	0.56	0.64	0.85	1.52	0.93
IsoButyric	3.91	0.08	0.002	0.99995	0.23	0.78	N/A	N/A	N/A	N/A
Butyric	4.20	0.08	0.001	0.99996	0.10	0.32	0.77	0.45	1.11	0.78
IsoValeric	4.42	0.09	0.001	0.99997	0.07	0.23	0.56	0.81	0.10	0.23
Valeric	4.78	0.91	0.000	0.99998	0.06	0.21	1.03	0.99	0.86	0.64
4-methyl valeric	5.12	0.09	0.001	0.99998	0.04	0.13	0.82	0.73	0.44	0.74
Hexanoic	5.34	1.02	0.001	0.99998	0.04	0.12	0.82	0.80	0.35	0.18
Heptanoic	5.89	1.05	0.003	0.99998	0.02	0.08	0.69	1.11	0.36	0.63

LOD—limit of detection; LOQ—limit of quantification; RSD—relative standard deviation; N/A—not available; SCFA—short-chain fatty acid.

**Table 2:** Percent (%) recovery at 100 and 1000 ppm spike levels on method blanks.

	Percent (%) Recovery at Respective Spike Levels ^1^	
	100 ppm	1000 ppm
Acetic	57.64 ± 2.89	54.24 ± 1.17
Propionic	87.60 ± 1.64	92.82 ± 1.43
IsoButyric	N/A	N/A
Butyric	100.71 ± 2.97	106.61 ± 1.54
IsoValeric	119.96 ± 4.10	127.83 ± 1.47
Valeric	121.41 ± 4.11	129.74 ± 1.88
4-methyl valeric	107.80 ± 26.30	129.52 ± 3.34
Hexanoic	124.74 ± 4.94	129.57 ± 9.63
Heptanoic	128.97 ± 5.43	140.94 ± 2.10

^1^ Values are unadjusted means ± SD; N/A—not available.

**Table 3:** Comparison of SCFAs extraction methods from fresh fecal samples.

SCFAs	AE Method Amount (µmol/g) ^1^	AE Method % RSD	TBME Method Amount (µmol/g) ^1^	TBME Method % RSD	*p*-Value AE vs. TBME ^2,3^
Acetic	69.42	8.63	36.80	20.94	<0.0001
Propionic	20.95	8.82	12.57	19.81	<0.0001
IsoButyric	5.89	6.82	3.53	19.45	<0.0001
Butyric	22.25	2.49	13.81	19.09	<0.0001
IsoValeric	8.48	6.05	5.24	20.21	<0.0001
Valeric	5.44	2.18	3.21	19.62	<0.0001
4-methyl valeric	ND	N/A	ND	N/A	N/A
Hexanoic	0.57	2.15	0.25	17.98	<0.0001
Heptanoic	ND	N/A	ND	N/A	N/A

*n* = 3; *n*—number of samples prepared by each extraction method. ^1^ Values are unadjusted means. ^2^ Student’s *t*-test. ^3^ Statistical significance at *p* < 0.05. SCFAs—short-chain fatty acids; AE—aqueous extraction; TBME—tert-butyl methyl ether extraction; RSD—relative standard deviation; ND—not detected; N/A—not applicable.

**Table 4:** Comparison of SCFAs content in lyophilized and fresh samples.

SCFAs	FD Amount ^1^ (µmol/g)	FR Amount ^1^ (µmol/g)	*p*-Value FD vs. FR ^2^
Acetic	73.8 ± 7.2	69.4 ± 5.2	0.504
Propionic	19.0 ± 1.7	21.0 ± 1.6	0.270
IsoButyric	4.8 ± 0.5	5.9 ± 0.3	0.058
Butyric	21.0 ± 2.8	22.2 ± 0.5	0.548
IsoValeric	7.0 ± 0.8	8.4 ± 0.4	0.101
Valeric	4.9 ± 0.7	5.4 ± 0.1	0.289
4-methyl valeric	0.3 ± 0.0	ND	1.62 × 10^−5^ *
Hexanoic	0.6 ± 0.0	0.6 ± 0.0	0.915
Heptanoic	0.3 ± 0.1	ND	0.011 *

*n* = 3; *n*—number of samples prepared for each pre-treatment type. ^1^ Values are unadjusted means ± SD. ^2^ Student’s *t*-test; * = *p* < 0.05. SCFAs—short-chain fatty acids; FD—lyophilized sample; FR—fresh sample; ND—not detected.

**Table 5:** Baseline demographics of study participants.

Variable	IR-Group (*n* = 20)	R-Group (*n* = 9)
Age (year) ^1^	34.8 ± 1.4	30.4 ± 1.7
BMI (kg/m^2^) ^1^	28.2 ± 0.8	23.4 ± 1.0
Female:Male (*n*)	9:11	6:3
Race (Asian:His:Cau:AA)	7:2:6:5	3:2:2:2

^1^ Values are unadjusted means ± SD. *n*—number of participants; BMI—body mass index; His—Hispanic; Cau—Caucasian; AA—African American; IR—insulin-resistant; R—reference.

**Table 6:** SCFAs before and after 4-week RRB or RRB + FOS intervention in the R- and IR-groups.

SCFAs	R-Group (*n* = 9)				IR-Group (*n* = 20)			
	Baseline (Week 0) Amount (µmol/g) ^1^	RRB (Week 4) Amount (µmol/g) ^1^	RRB + FOS (Week 4) Amount (µmol/g) ^1^	*p*-Value Interv at Week 4 ^2^	Baseline (Week 0) Amount (µmol/g) ^1^	RRB (Week 4) Amount (µmol/g) ^1^	RRB + FOS (Week 4) Amount (µmol/g) ^1^	*p*-Value Interv at Week 4 ^2^
Acetic	178.7 ± 26.0	159.7 ± 29.2	155.3 ± 35.1	0.905	162.0 ± 22.9	142.3 ± 18.4	164.6 ± 17.7	0.257
Propionic	72.6 ± 12.9	53.7 ± 8.3	51.5 ± 9.3	0.712	65.7 ± 11.7	52.9 ± 5.9	62.1 ± 10.5	0.426
IsoButyric	6.6 ± 1.7	6.2 ± 1.1	5.2 ± 0.8	0.412	6.1 ± 0.7	5.8 ± 0.6	4.9 ± 0.5	0.208
Butyric	62.2 ± 12.9	52.4 ± 14.5	48.7 ± 7.9	0.781	52.9 ± 7.8	45.3 ± 4.9	42.6 ± 3.9	0.640
IsoValeric	10.5 ± 3.0	9.3 ± 1.7	7.7 ± 1.2	0.394	9.0 ± 1.0	8.7 ± 0.9	6.9 ± 0.7	0.097
Valeric	7.7 ± 1.7	7.3 ± 1.7	6.1 ± 1.3	0.438	6.9 ± 1.1	5.4 ± 0.7	5.3 ± 0.7	0.918
4-Methyl Valeric	0.5 ± 0.3	0.1 ± 0.1	0.3 ± 0.1	0.040 *	0.3 ± 0.1	0.3 ± 0.1	0.2 ± 0.1	0.827
Hexanoic	1.8 ± 0.7	1.0 ± 0.4	2.1 ± 0.8	0.224	1.5 ± 0.5	1.2 ± 0.5	1.3 ± 0.4	0.886
Heptanoic	0.7 ± 0.2	0.1 ± 0.1	0.5 ± 0.2	0.073	1.0 ± 0.7	0.2 ± 0.1	0.4 ± 0.1	0.352
Total SCFA	341.4 ± 51.5	289.7 ± 53.4	277.3 ± 50.6	0.827	305.3 ± 40.3	262.1 ± 28.4	288.3 ± 28.9	0.442

^1^ Values are unadjusted means ± SEM. ^2^ ANCOVA Analysis; * *p* < 0.05. *n*—number of participants; RRB—red raspberry smoothie; RRB + FOS—red raspberry smoothie with fructo-oligosaccharide; Interv—intervention; SCFAs—short-chain fatty acids; IR—insulin-resistant; R—reference.

## Data Availability

The data in this study are available upon request to the corresponding author. Data is not publicly available due to privacy.

## Supplementary Materials

The following supporting information can be downloaded at: https://www.mdpi.com/article/10.3390/metabo13111106/s1. Figure S1: Overlay of chromatograms from samples prepared via AE method and TBME method. Figure S2: Overlay of chromatograms from samples pre-treated by freeze-drying or left fresh and extracted by AE method.

## References

[1] Bergman E.N. (1990). Energy contributions of volatile fatty acids from the gastrointestinal tract in various species. Physiol. Rev..

[2] Niccolai E., Baldi S., Ricci F., Russo E., Nannini G., Menicatti M., Poli G., Taddei A., Bartolucci G., Calabrò A.S. (2019). Evaluation and comparison of short chain fatty acids composition in gut diseases. World J. Gastroenterol..

[3] den Besten G., van Eunen K., Groen A.K., Venema K., Reijngoud D.J., Bakker B.M. (2013). The role of short-chain fatty acids in the interplay between diet, gut microbiota, and host energy metabolism. J. Lipid Res..

[4] Rasmussen H.S., Holtug K., Mortensen P.B. (1988). Degradation of amino acids to short-chain fatty acids in humans. An in vitro study. Scand. J. Gastroenterol..

[5] Jeon B., Choi O., Um Y., Byoung-In S. (2016). Production of medium-chain carboxylic acids by Megasphaera sp. MH with supplemental electron acceptors. Biotechnol. Biofuels.

[6] Ferry J.G. (2011). Acetate kinase and phosphotransacetylase. Methods Enzymol..

[7] Isipato M., Dessì P., Sánchez C., Mills S., Ijaz U.Z., Asunis F., Spiga D., De Gioannis G., Mascia M., Collins G. (2020). Propionate Production by Bioelectrochemically-Assisted Lactate Fermentation and Simultaneous CO_2_ Recycling. Front. Microbiol..

[8] Frampton J., Murphy K.G., Frost G., Chambers E.S. (2020). Short-chain fatty acids as potential regulators of skeletal muscle metabolism and function. Nat. Metab..

[9] Primec M., Mičetić-Turk D., Langerholc T. (2017). Analysis of short-chain fatty acids in human feces: A scoping review. Anal. Biochem..

[10] Hoving L.R., Heijink M., van Harmelen V., van Dijk K.W., Giera M. (2018). GC-MS Analysis of Short-Chain Fatty Acids in Feces, Cecum Content, and Blood Samples. Methods Mol. Biol..

[11] Scortichini S., Boarelli M.C., Silvi S., Fiorini D. (2020). Development and validation of a GC-FID method for the analysis of short chain fatty acids in rat and human faeces and in fermentation fluids. J. Chromatogr. B Analyt. Technol. Biomed. Life Sci..

[12] Zhang S., Wang H., Zhu M.J. (2019). A sensitive GC/MS detection method for analyzing microbial metabolites short chain fatty acids in fecal and serum samples. Talanta.

[13] Han J., Lin K., Sequeira C., Borchers C.H. (2015). An isotope-labeled chemical derivatization method for the quantitation of short-chain fatty acids in human feces by liquid chromatography-tandem mass spectrometry. Anal. Chim. Acta.

[14] Liebisch G., Ecker J., Roth S., Schweizer S., Öttl V., Schött H.F., Yoon H., Haller D., Holler E., Burkhardt R. (2019). Quantification of Fecal Short Chain Fatty Acids by Liquid Chromatography Tandem Mass Spectrometry-Investigation of Pre-Analytic Stability. Biomolecules.

[15] Zhang X., Zhao A., Sandhu A.K., Edirisinghe I., Burton-Freeman B.M. (2020). Functional Deficits in Gut Microbiome of Young and Middle-Aged Adults with Prediabetes Apparent in Metabolizing Bioactive (Poly)phenols. Nutrients.

[16] Zhang X., Zhao A., Sandhu A.K., Edirisinghe I., Burton-Freeman B.M. (2022). Red Raspberry and Fructo-Oligosaccharide Supplementation, Metabolic Biomarkers, and the Gut Microbiota in Adults with Prediabetes: A Randomized Crossover Clinical Trial. J. Nutr..

[17] Hsu Y.L., Chen C.C., Lin Y.T., Wu W.K., Chang L.C., Lai C.H., Wu M.S., Kuo C.H. (2019). Evaluation and Optimization of Sample Handling Methods for Quantification of Short-Chain Fatty Acids in Human Fecal Samples by GC-MS. J. Proteome Res..

[18] Eberhart B.L., Wilson A.S., O’Keefe S.J.D., Ramaboli M.C., Nesengani L.T. (2021). A simplified method for the quantitation of short-chain fatty acids in human stool. Anal. Biochem..

[19] Zhao G., Nyman M., Jönsson J.A. (2006). Rapid determination of short-chain fatty acids in colonic contents and faeces of humans and rats by acidified water-extraction and direct-injection gas chromatography. Biomed. Chromatogr..

[20] Verbeke K.A., Boobis A.R., Chiodini A., Edwards C.A., Franck A., Kleerebezem M., Nauta A., Raes J., van Tol E.A., Tuohy K.M. (2015). Towards microbial fermentation metabolites as markers for health benefits of prebiotics. Nutr. Res. Rev..

[21] Bravo J.A., Julio-Pieper M., Forsythe P., Kunze W., Dinan T.G., Bienenstock J., Cryan J.F. (2012). Communication between gastrointestinal bacteria and the nervous system. Curr. Opin. Pharmacol..

[22] Wen L., Ley R.E., Volchkov P.Y., Stranges P.B., Avanesyan L., Stonebraker A.C., Hu C., Wong F.S., Szot G.L., Bluestone J.A. (2008). Innate immunity and intestinal microbiota in the development of Type 1 diabetes. Nature.

[23] de la Cuesta-Zuluaga J., Mueller N.T., Álvarez-Quintero R., Velásquez-Mejía E.P., Sierra J.A., Corrales-Agudelo V., Carmona J.A., Abad J.M., Escobar J.S. (2018). Higher Fecal Short-Chain Fatty Acid Levels Are Associated with Gut Microbiome Dysbiosis, Obesity, Hypertension and Cardiometabolic Disease Risk Factors. Nutrients.

[24] Weitkunat K., Schumann S., Nickel D., Kappo K.A., Petzke K.J., Kipp A.P., Blaut M., Klaus S. (2016). Importance of propionate for the repression of hepatic lipogenesis and improvement of insulin sensitivity in high-fat diet-induced obesity. Mol. Nutr. Food Res..

[25] Gao Z., Yin J., Zhang J., Ward R.E., Martin R.J., Lefevre M., Cefalu W.T., Ye J. (2009). Butyrate improves insulin sensitivity and increases energy expenditure in mice. Diabetes.

[26] Chung W.S.F., Meijerink M., Zeuner B., Holck J., Louis P., Meyer A.S., Wells J.M., Flint H.J., Duncan S.H. (2017). Prebiotic potential of pectin and pectic oligosaccharides to promote anti-inflammatory commensal bacteria in the human colon. FEMS Microbiol. Ecol..

[27] Zheng C., Liu R., Xue B., Luo J., Gao L., Wang Y., Ou S., Li S., Peng X. (2017). Impact and consequences of polyphenols and fructooligosaccharide interplay on gut microbiota in rats. Food Funct..

[28] Alles M.S., Hautvast J.G., Nagengast F.M., Hartemink R., Van Laere K.M., Jansen J.B. (1996). Fate of fructo-oligosaccharides in the human intestine. Br. J. Nutr..

[29] Scholtens P.A., Alles M.S., Willemsen L.E., van den Braak C., Bindels J.G., Boehm G., Govers M.J. (2006). Dietary fructo-oligosaccharides in healthy adults do not negatively affect faecal cytotoxicity: A randomised, double-blind, placebo-controlled crossover trial. Br. J. Nutr..

[30] Tamanai-Shacoori Z., Smida I., Bousarghin L., Loreal O., Meuric V., Fong S.B., Bonnaure-Mallet M., Jolivet-Gougeon A. (2017). *Roseburia* spp.: A marker of health?. Future Microbiol..

[31] Clausen M.R., Mortensen P.B. (1995). Kinetic studies on colonocyte metabolism of short chain fatty acids and glucose in ulcerative colitis. Gut.

[32] Cummings J.H., Pomare E.W., Branch W.J., Naylor C.P., Macfarlane G.T. (1987). Short chain fatty acids in human large intestine, portal, hepatic and venous blood. Gut.

