# Supplementary material for: An Improved Validated Method for the Determination of Short-Chain Fatty Acids in Human Fecal Samples by Gas Chromatography with Flame Ionization Detection (GC-FID)
Source: Metabolites. 2023 Oct 24;13(11):1106. doi: 10.3390/metabo13111106 (PMC10673161; doi:10.3390/metabo13111106)
Supplement: Supplementary file 1 [file metabolites-13-01106-s001.zip › metabolites-2596671-supplementary.pdf]

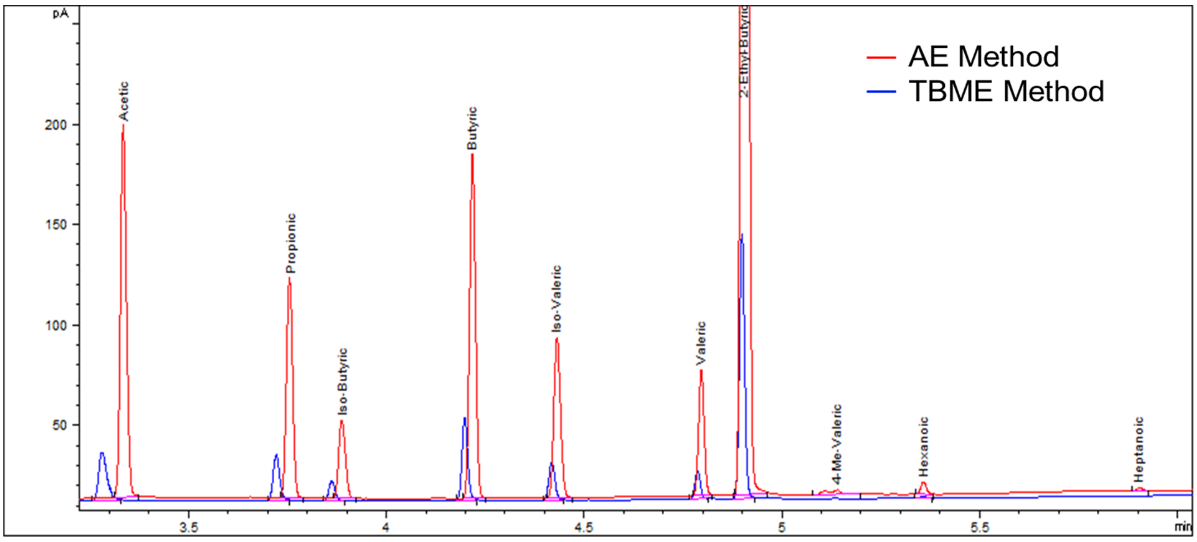

Figure S1. Overlay of chromatograms from samples prepared via AE method and TBME method.

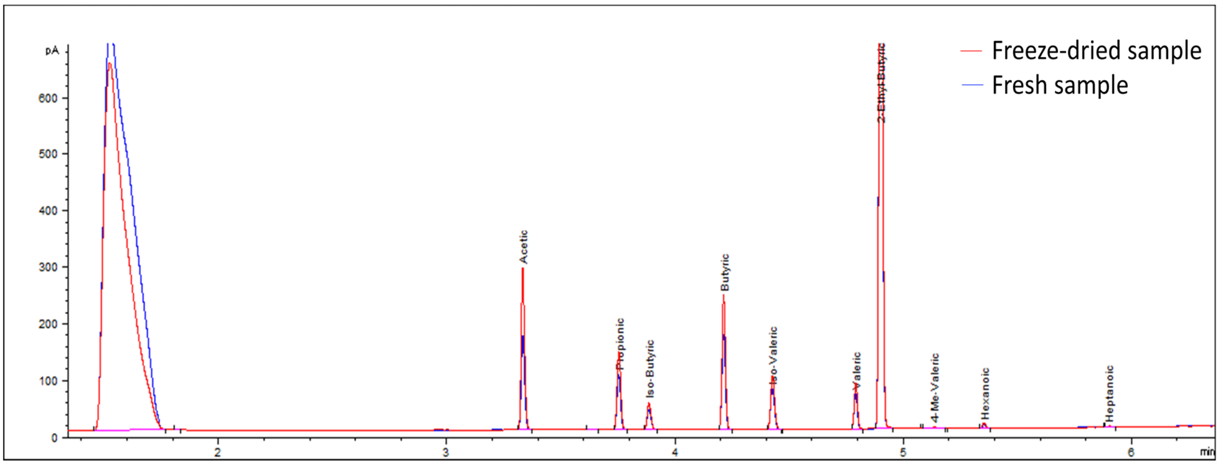

Figure S2. Overlay of chromatograms from samples pre-treated by freeze-drying or left fresh and extracted by AE method.
